# A Long-Acting Curcumin Nanoparticle/In Situ Hydrogel Composite for the Treatment of Uveal Melanoma

**DOI:** 10.3390/pharmaceutics13091335

**Published:** 2021-08-25

**Authors:** Lingxiao Xie, Weizhou Yue, Khaled Ibrahim, Jie Shen

**Affiliations:** 1 Department of Biomedical and Pharmaceutical Sciences, University of Rhode Island, Kingston, RI 02881, USA; lingxiao_xie@uri.edu (L.X.); weizhou_yue@uri.edu (W.Y.); khaled_ibrahim@uri.edu (K.I.); 2 Department of Chemical Engineering, University of Rhode Island, Kingston, RI 02881, USA

**Keywords:** in situ hydrogel, nanoparticle/hydrogel composite, sustained delivery, curcumin, uveal melanoma

## Abstract

Uveal melanoma (UM) is the most common primary intraocular tumor in adults with high mortality. In order to improve prognosis and survival of UM patients, it is critical to inhibit tumor progression and metastasis as early as possible after the initial presentation/diagnosis of the disease. Sustained local delivery of antitumor therapeutics in the posterior region can potentially achieve long-term UM inhibition, improve target therapeutic delivery to the posterior segments, as well as reduce injection frequency and hence improved patient compliance. To address the highly unmet medical need in UM therapy, a bioinspired in situ gelling hydrogel system composed of naturally occurring biopolymers collagen and hyaluronic acid was developed in the present research. Curcumin with anti-cancer progression, anti-metastasis effects, and good ocular safety was chosen as the model therapeutic. The developed in situ gelling delivery system gelled at 37 °C within two minutes and demonstrated excellent biocompatibility and slow degradation. The curcumin-loaded nanoparticle/hydrogel composite was able to sustain release payload for up to four weeks. The optimized nanoparticle/hydrogel composite showed effective inhibition of human UM cell proliferation. This novel nanoparticle/in situ hydrogel composite demonstrated a great potential for the treatment of the rare and devastating intraocular cancer.

## 1. Introduction

Uveal melanoma (UM) is considered a rare disease yet the most common primary intraocular tumor with high metastasis and lethality in adults [1,2]. Treatment modalities including globe-preserving therapies (e.g., proton beam radiotherapy, plaque brachytherapy) and enucleation have been commonly used. Despite the aggressive treatment strategies, a high recurrence rate, in particular following transscleral resection (up to 32.6%), has remained a challenge [3,4,5]. Moreover, nearly a half of UM patients eventually developed metastatic diseases (primarily in the liver) that are resistant to systemic chemo- and immunotherapy, leading to poor prognosis and high mortality [6]. Up to now, there has been a lack of effective treatment to prevent UM metastasis. Accordingly, it is considered that early detection and intervention may be critical to improve prognosis and survival of UM.

The uvea is one of the most capillary-rich tissues of the body. Most UM occurs in the posterior segments such as choroid (~90%), iris (~5%) and ciliary body (~5%) [7,8]. Due to the lack of lymphatic vessels in the choroid, primary UM metastasizes almost exclusively via the hematogenous route [9,10]. As a result, effective suppression of molecular mechanisms that activate angiogenesis, tumor cell mobility and invasion, has demonstrated the potential in improving UM therapy [11,12,13]. For example, regulator or knockdown hypoxia-response regulating genes cyclic AMP-response-element binding protein and hypoxia-inducible factors-1 (HIF-1), vascular endothelial growth factor (VEGF) can markedly decreased UM cell or tumor growth [14,15,16,17,18]. However, the presence of anatomical ocular barriers (e.g., cornea, blood-retina barrier) and physiological constraints (e.g., nasolacrimal drainage, limited capacity in the cul-de-sac of the eye) prevents about 99% of drug administrated systemically and topically to reach the posterior segments [19]. Local delivery of therapeutics in the posterior chamber (e.g., via intravitreal administration) can circumvent these barriers to directly target the tumor in the posterior chamber to improve anti-UM effect while reducing off-target toxicity. As most therapeutics require frequent dosing due to their short half-lives in the vitreous humor, a long-acting local delivery platform that can sustained deliver therapeutics in the posterior chamber is considered desirable to improve therapeutic effect and patient compliance for the UM treatment.

Various nanocarriers/microcarriers with sustained drug delivery capability have been developed in recent years for ocular use [20,21]. Due to their size, and flexible surface functionality, nanocarriers/microcarriers offer advantages in drug delivery for the treatment of posterior eye diseases [22,23]. However, there are disadvantages associated with nanocarriers/microcarriers directly administrated in the posterior chamber, including potential toxicity due to high burst drug release; rapid phagocytic clearance which may decrease therapeutic effect; particle aggregation due to large surface area; and particle migration which may cause ocular inflammation [23]. Currently, marketed long-acting intravitreal implant drug products (e.g., Ozudex^®^, IIuvien^®^) are able to sustain deliver therapeutics in the posterior segments over weeks to months. These implant products are administrated via either an injector/applicator with a large size needle (i.e., 22-gauge) or surgical procedures and hence poor patient compliance. Over the past decade, in situ gelling hydrogels that can be injected as solutions via fine needles (i.e., 30-gauge or smaller) and transform to gels/depots at the injection site, have attracted increasing interests due to excellent injectability and improved patient compliance [24,25,26]. However, the use of synthetic polymer-based hydrogels (e.g., polystyrene, poloxamer) has limited its applications due to concerns on biodegradability and potentially triggering inflammatory response and influencing retinal function [25,27]. Moreover, a crosslinking initiator or catalyst (such as a metal catalyst, or photoinitiator) or ultraviolet (UV) light is often needed to facilitate polymer crosslinking and gelation, which raises biocompatibility concerns [28,29,30]. Accordingly, it is essential to develop a biodegradable and biocompatible in situ forming hydrogel platform that gels within seconds to minutes and is capable of releasing payload over weeks to months.

The vitreous body is mainly composed of hyaluronic acid and different types of collagens, primarily type II collagen [31,32]. Inspired by the natural components of the vitreous body, an in situ gelling hydrogel delivery platform composed of naturally occurring biopolymers such as hyaluronic acid (HA) and collagen (type II) was developed to sustained release payload for UM therapy in the present research. The in situ gelling hydrogel system provides easy administration, prolongs retention time, and prevents drug/particle migration. Curcumin with good ocular safety was chosen as the model therapeutic. It has been reported curcumin can regulate hypoxia-inducible factors-1α (HIF-1α) in hepatocellular carcinoma cells, pituitary adenomas, and inhibits angiogenesis in zebrafish [33,34,35,36]. Curcumin also showed anti-proliferation and down-regulation of the expression of genes involved in apoptosis, cell proliferation and transformation against various cancers (e.g., breast cancer, lung cancer, colorectal cancer) [37,38,39,40,41]. Moreover, curcumin has been shown to induce cell death in human UM cells via the mitochondrial pathway [42]. As curcumin is poorly water soluble, curcumin-loaded nanoparticles with a hydrophilic coating layer were fabricated to improve its vitreous transport and cellular uptake. The curcumin-loaded polymeric nanoparticles were incorporated in the collagen II and HA hydrogel (CO–HA Gel) matrix. The rheological properties, payload release as well as in vitro anti-UM effect of the developed curcumin nanoparticle/in situ hydrogel composites were investigated.

## 2. Materials and Methods

### 2.1. Materials

Collagen (type II) from calf articular joints was purchased from Elastic Products Company, Inc (Owensville, MO). Sodium hyaluronate (HA, molecular weight (MW): 15 MDa) was purchased from Lifecore Biomedical (Chaska, MN, USA). Eight-arm polyethylene glycol (PEG) succinimidyl glutarate (tripentaerythritol) (8-arm PEG, MW: 20 kDa and 40 kDa) were purchased from JenKem Technology USA (Plano, TX, USA). Picrylsulfonic acid solution (TNBS), polysorbate (Tween) 80, N-acetylcysteine (NAC), butylated hydroxytoluene (BHT), glycine (ReagentPlus^®^, ≥99%), dichloromethane (DCM, ACS reagent, ≥99.5%), poly(vinyl alcohol) (PVA, 87–90% hydrolyzed, MW: 30–70 kDa), sucrose (≥99.5%), collagenase from clostridium histolyticum (1.5 kU), hyaluronidase (type II) from sheep testes, dimethyl sulfoxide (DMSO, ACS reagent, ≥99.9%), acetonitrile (HPLC grade, ≥99.9%), trifluoroacetic acid (TFA, HPLC grade, ≥99.0%) were purchased from Sigma-Aldrich (St. Louis, MO, USA). Curcumin was purchased from TCI Chemicals (Portland, OR, USA). Methoxypoly(ethylene glycol)-b-poly(lactide-co-glycolide) (mPEG-PLGA, L/G: 50/50, *w*/*w*; MW: 5 kDa/55 kDa) was purchased from PolySciTech^®^, Akina, Inc. (West Lafayette, IN, USA). LIVE/DEAD^TM^ viability/cytotoxicity kit (for mammalian cells), CyQUANT™ MTT cell viability assay, phosphate-buffered saline (PBS, pH 7.4), trypsin-EDTA (0.25%) phenol red, 4-(2-hydroxyethyl)-1-piperazineethanesulfonic acid (HEPES, 1 M) and penicillin-streptomycin (10,000 U/mL) were purchased from Thermo Fisher Scientific (Waltham, MA). RPMI-1640 medium (ATCC^®^ 30-2001™) and fetal bovine serum (FBS) (ATCC^®^ 30-2020™) were purchased from ATCC (Manassas, VA, USA).

Human uveal melanoma (UM) cell line MP-38 (ATCC^®^ CRL-3296) was grown and maintained in RPMI-1640 media with 20% (*v*/*v*) FBS and 1% (*v*/*v*) penicillin-streptomycin. Cells were cultured in an incubator supplied with 5% CO_2_ at 37 °C. The culture medium was replaced every 2–3 days.

### 2.2. Preparation of Curcumin-Loaded Nanoparticles

Curcumin-loaded polymeric nanoparticles (Cur NP) were prepared via an emulsion-solvent-evaporation method as previously reported [43]. Briefly, curcumin and mPEG-PLGA (curcumin/mPEG-PLGA = 1:7.5, *w*/*w*) were dissolved in 2 mL of DCM. The organic phase was added into 10 mL of an aqueous phase containing 1% (*w*/*v*) PVA to form a primary emulsion. Following size reduction via probe sonication, DCM was removed by evaporation and the residual solvent was further removed under reduced pressure. The resultant NP suspension was then filtered using a 0.22 µm filter (polytetrafluoroethylene, PTFE). NP was purified via ultra-centrifugation at 229,600× *g* for 1 h with distilled (DI) water three times. The concentrated NP suspension was then lyophilized using sucrose as the cryoprotectant (sucrose/NP: 25/1, *w*/*w*). The lyophilized NP was stored at 4 °C until further use.

### 2.3. Characterization of Cur NP

Lyophilized Cur NP was accurately weighed, and curcumin was extracted using 200 µL of DMSO followed by acetonitrile dilution to 2 mL. The sample was then filtered using a 0.22 μm syringe filter (PTFE) and curcumin content was determined via HPLC (Hitachi) with a ZORBAX Eclipse XDB C18 column (5 µm, 250 × 4.6 mm) (Agilent Technologies, Inc. Santa Clara, CA, USA). The HPLC mobile phase consisted of 0.1% TFA in water and acetonitrile (40/60, *v*/*v*) with a flow rate of 1 mL/minute. The effluent was monitored at the wavelength of 420 nm. The retention of curcumin was around 5.2 min. The detection and quantification limits of the HPLC analytical method were 0.027 and 0.083 µg/mL, respectively. Curcumin loading was calculated using the following Equation (1):

(1) $$Dug\;loading\;\left(\%\right)=\frac{Curcumin\;amount\;in\;nanoparticles}{Lyophilized\;nanoparticle\;weight}\times 100$$

The particle size and size distribution (polydispersity index, PDI), and surface charge of NP before and after lyophilization were characterized using Zetasizer Nano ZS90 (Malvern Instruments Ltd., Malvern, UK). The backscattering angel was 173° with a standard laser wavelength *λ* of 633 nm. The measurement of dynamic diameter (D_H_) is according to the Stokes–Einstein equation as a function of the diffusion coefficient (D), temperature (T), and viscosity (η). The measurements were carried out in triplicate.

### 2.4. Fabrication of CO–HA Gels and Composites

A HA solution in PBS (10 mM, pH 7.4) (5 mg/mL) was mixed with a collagen II solution in a phosphate buffer (20 mM, pH 7.4) (5 mg/mL) at an optimized volume ratio of 3/1 (HA/collagen). An 8-arm PEG solution in PBS (10 mM, pH 7.4) (200 mg/mL) was then added into the mixture at a volume ratio of 1/13.5, and the blank hydrogel (CO–HA Gel) was formed when incubating the mixture at 37 °C.

To form NP/CO–HA Gel composite, accurately weighed NP (either blank or Cur NP) was added into the collagen II solution prior to mixing with other gel components (i.e., HA and PEG). The NP/CO–HA Gel composite was then formed following the similar procedure described above for the blank CO–HA Gel. To form curcumin/hydrogel composite (Cur/CO–HA Gel), a curcumin solution in ethanol (2.424 mg/mL) was mixed with the gel components (i.e., collagen II, HA and PEG) and the mixture was then incubated at 37 °C.

### 2.5. Rheological Study of Blank CO–HA Gels and NP/CO–HA Gel Composites

Rheological properties of blank CO–HA Gel composed of different MW PEG with or without NP were determined using a TA Discovery HR-2 rheometer (TA Instruments, New Castle, DE) with a cone shape geometry (40 mm, 2°). After mixing three gel components (i.e., collagen, HA and PEG) at the ratios described in Section 2.4, 800 μL of the mixture was immediately loaded onto a sample loading plate pre-equilibrated at 25 °C. The cone shape geometry was lowered to a pre-determined gap (48 µm) to obtain a vertical side wall. Oscillation amplitude analysis was performed at 37 °C to determine a linear viscoelastic region (LVR) at a frequency of 1 Hz. Gelling time of hydrogels was determined via time sweep with 1 Hz frequency and 5% strain in 20 min at 37 °C. Storage modulus of the CO–HA Gel was obtained by unconfined compression measurements with oscillation frequency ranging from 0.1 to 10 Hz at 5% strain at 37 °C. The CO–HA Gel and NP/CO–HA Gel composites were placed between a solid plate and the rheometer geometry, and freely expanded laterally during the dynamic compression. The measurements were carried out in triplicate.

### 2.6. Crosslinking Density Study

The crosslinking degree of the CO–HA Gel was calculated based on the rubber elasticity theory using experimental storage modulus G’ as reported previously [44]. The number of elastically active junctions in the network per unit of volume (*n_e_*, mol/m^3^) was calculated using the following Equation (2):

(2) $$G^{\prime }=n_{e}RT$$

where *G*′ is storage modulus, *R* is the gas constant, and *T* is the absolute temperature.

### 2.7. Determination of Swelling Property

Disc-shaped CO–HA Gel samples were formed in a 96-well plate in triplicate. The sample was transferred into an Eppendorf tube and weighed on a microscale (Mettler Toledo, Greifensee, Switzerland) to obtain the initial weight (*W_i_*). The sample was then immersed in saline and incubated at 37 °C to allow free swelling for 48 h. The sample was taken out and excess saline was gently removed. Fully swollen samples (*W_s_*) were weighed on a microscale and lyophilized (FreeZone Triad Cascade Benchtop Freeze Dryer, LABCONCO, Kansas City, MO, USA). Hydrogel swelling was calculated based on mass change percentage (%) using the following equation:

(3) $$Mass\;increase\;\left(\%\right)=\frac{W_{S}-W_{i}}{W_{i}}\times 100\%$$

The weight (*W_d_*) of the freeze-dried hydrogels was also determined, and the equilibrium water content (*EWC*) was calculated using the following equation:

(4) $$EWC=\frac{W_{s}-W_{d}}{W_{s}}$$

### 2.8. Morphology/Internal Structure of Cur NP and NP/CO–HA Gel Composites

Lyophilized Cur NP was transferred to a metal substrate with double-sided carbon tape. A thin film of Au was sputtered coated onto the NP for 30 s. The sample was then imaged using SIGMA VP Field Emission-scanning electron microscope (FE-SEM) (ZEISS, Oberkochen, Germany). Hydrogel samples were imaged via cryo-SEM. The blank CO–HA Gel sample was formed and frozen in liquid nitrogen and attached to the specimen holder of a cryo-transfer system. The sample was transferred onto a cryostage in the SEM system and cross-sectioned, and subsequently transferred to a SEM sample stage and examined via FE-SEM. The structure of Cur NP/CO–HA Gel composite was also examined using cryo-transmission electron microscopy (cryo-TEM). Small pieces of Cur NP/CO–HA Gel composite were transferred on a TEM copper grid. The grid was quickly immersed in liquid ethane. The frozen-hydrated specimens were mounted on a cryo-holder and observed using JEM-2100 TEM (JEOL USA, Inc, Peabody, MA, USA). The obtained images were analyzed by image J find edge process and threshold. The % of mesh area and mesh size were analyzed by area fraction and ferret’s diameter function, respectively. Mesh size diameters *D*_90_, *D*_50_, and *D*_10_ were obtained by excel percentile function. Diameter span was calculated using the following equation:

(5) $$Span=\frac{D_{90}-D_{10}}{D_{50}}$$

### 2.9. In Vitro Degradation Study

In vitro degradation of CO–HA Gels was investigated by incubating formed hydrogels (300 μL) in 300 μL of saline at 37 °C in triplicate. At pre-determined time intervals, the hydrogels were removed and gently washed with DI water. The remaining free amine groups in the hydrogel were determined using a spectrophotometric method [45,46]. Briefly, 187 µL of 0.1% (*w*/*v*) TNBS diluted with 0.1 M NaHCO_3_ were added into the hydrogel samples and incubated for 2 h at 37 °C. The reaction was ceased by adding 563 µL of 6 N HCl to the samples followed by autoclave for 1 h at 120 °C. After the samples were cooled down to room temperature, the mixture was then spectrophotometrically measured at an absorbance wavelength of 340 nm using a microplate-reader (Spectramax M2 Multi-Mode microplate reader, Molecular Devices, LLC, San Jose, CA, USA). The free amine group content was calculated based on a standard curve prepared using glycine standards. The total free amine group content (*Con_Total_*) was determined using the same amount of collagen II prior to the degradation study. Percentage (%) of free amine groups in the formed hydrogel was calculated based on the Equation (6):

(6) $$Free\;amine\;group\;\left(\%\right)=\frac{Con_{Sample}}{Con_{Total}}\times 100\%$$

In vitro degradation of CO–HA Gels (300 μL) was also studied in the presence of enzymes, including 22.5 U (75 U/mL) collagenase and 5 U (16.7 U/mL) hyaluronidase in 300 µL of saline at 37 °C in triplicate. The hydrogels incubated in saline without the presence of enzymes at 37 °C were studied as control group.

### 2.10. In Vitro Release Study of Cur NP/CO–HA Gel Composites

In vitro curcumin release characteristics from Cur NP/CO–HA Gel composites were studied in triplicate. The composite (200 µL) was immersed in 200 µL of saline containing 10% (*w*/*v*) Tween 80, 0.1% (*w*/*v*) NAC, and 0.01% (*w*/*v*) BHT, and incubated at 37 °C in a water bath with shaking at 45 rpm. At pre-determined time points, 150 µL of release media was withdrawn and replenished with fresh media. The released curcumin amount was analyzed by HPLC. The percentage of cumulative curcumin released was calculated.

### 2.11. In Vitro Anti-UM Study of Cur NP/CO–HA Gel Composites

Anti-UM study of Cur NP/CO–HA Gel composite was conducted by using transwell insert (6.5 mm diameter with 8.0 µm pore polyester membrane, Corning Life Sciences, Corning, NY, USA). MP-38 cells were seeded in a 24-well plate (8 × 10^4^ cells per well) and incubated for 24 h till confluency reached around 80%. Cur NP/Gel 1, BLK NP/Gel 1, Cur/Gel 1 and Cur NP suspension (curcumin concentration: 201.0 µg/mL) were tested. The gel mixture (100 µL) was transferred into a transwell insert chamber and incubated in a 37 °C incubator to form the composites. The insert was then placed in the 24-well plate seeded with MP-38 cells and completely immersed in the culture medium. For the Cur NP suspension group, 100 µL of suspension (without hydrogel components) was added into a transwell insert before the insert was placed into a 24-well plate. The cells seeded on the 24-well plate with the transwell inserts containing different treatments were then cultured at 37 °C for 3 days. Following the treatment, the transwell inserts were removed and cells were washed with PBS three times. CyQUANT^®^ MTT reagents (Thermo Fisher Scientific, Waltham, MA, USA) were prepared and added into the 24-well plate following the manufacturer’s protocol. The absorbance of the samples at 570 nm was determined using a microplate reader. MP-38 cells without any treatment grown in the RPMI-1640 medium were studied as control. Cell viability was calculated based on the equation below:

(7) $$Cell\;viability\;\left(\%\right)=\left(\frac{OD_{sample}}{OD_{Control}}\right)\times 100\%$$

In order to further demonstrate the anti-UM effect was due to the sustained curcumin release from the NP/CO–HA Gel composites, MP-38 cells were directly cultured on the surface of the CO–HA Gels and BLK NP/CO–HA Gel composites. MP-38 cells directly grown in a culture plate without Gels was studied as control. Tested composites (40 µL) were mixed at the ratios described in Section 2.4 and transferred into a 96-well plate. The 96-well plate was incubated at 37 °C for 30 min. After the CO–HA Gel and composites were well formed, 150 µL of RPMI-1640 medium without FBS was added into the wells and incubated for 2 h at 37 °C. The medium was then removed and replaced with 150 µL of fresh RPMI-1640 medium contained 20% FBS and 1% penicillin-streptomycin before seeding MP-38 cells (1.5 × 10^4^ cell per well) on the surface of the CO–HA Gel or blank NP/CO–HA Gel composites. At pre-determined timepoints, cells were stained with LIVE/DEAD assay following the manufacturer’s instruction and imaged by EVOS^®^ FL Auto Cell Imaging System (Thermo Fisher Scientific, Waltham, MA, USA) via GFP, TexasRed, and Transmission channels. Cell number was quantified by Image J. Cell viability was calculated based on the equation below:

(8) $$Cell\;viability\;\left(\%\right)=\left(\frac{Cell\;number_{Live\;\left(GFP\right)}}{Cell\;number_{Live\;\left(GFP\right)}+Cell\;number_{Dead\;\left(TxRed\right)}}\right)\times 100\%$$

### 2.12. Statistical Analysis

All data was collected and presented as mean ± S.D. (standard deviation). Statistical analysis was performed using Student’s *t*-test with *p* < 0.05 as the minimal level of significance.

## 3. Results and Discussion

### 3.1. Characteristics of NP

As shown in Table 1, NP with (Cur NP) or without curcumin (BLK NP) was 161.9 ± 5.1 nm and 156.6 ± 1.5 nm in size with a narrow particle size distribution (PDI < 0.1), respectively. BLK and Cur NP were negatively charged with a zeta potential around −26 to −28 mV. Due to the abundant presence of negatively charged HA in the vitreous chamber, the negative surface charge of Cur NP was considered to be favorable, as it can avoid charge trapping and maintain NP mobility to facilitate drug delivery to the target posterior segments (e.g., choroid).

**Figure 1:**
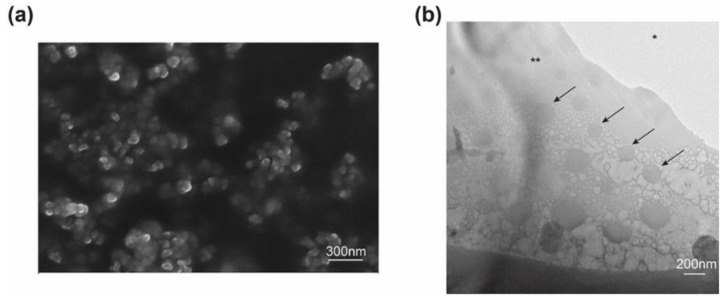
(**a**) Scanning electron microscope (SEM) image of curcumin nanoparticles (Cur NP). (**b**) Representative cryogenic transmission electron microscope (Cryo-TEM) image of the Cur NP/hydrogel composite. Arrows indicate Cur NP; * TEM cooper grid, ** Gel composite.

In order to ensure long-term storage stability of Cur NP and to fabricate NP/CO–HA Gel composites, dry NP was obtained via lyophilization. During the lyophilization process, the pre-frozen step induced water crystal that can disrupt the stabilizer shell around NP, resulting in clustering during the reconstitution step [47]. Accordingly, various cryoprotectants (i.e., trehalose, mannitol, and sucrose) at different cryoprotectant to NP weight ratios were investigated. Among all the cryoprotectants studied, sucrose demonstrated superior cryoprotective effect against nanoparticle aggregation during the lyophilization process and hence was selected as the cryoprotectant. As shown in Table 1, NP remained similar particle size and zeta potential following the reconstitution process when sucrose was used at a NP/sucrose weight ratio of 25:1. Curcumin loading of the lyophilized Cur NP was 0.20 ± 0.004% (*w*/*w*) (*n* = 3). The reported IC_50_ of curcumin NP against cancer and metastatic cancer cells (e.g., pancreatic, ovarian, metastatic breast) was in the range of 10–15 µM [48,49]. Therefore, it is considered that our NP had sufficient curcumin loading. In addition, the value IC_50_ of Cur NP against MP-38 cells was about 19% lower than that of the first-line treatment dacarbazine group (data not shown). The morphology of lyophilized Cur NP was shown in Figure 1a. The NP showed a spherical shape with a homogenous size distribution (around 85.8 ± 15.6 nm). It was expected that the particle size observed via SEM was smaller than the hydrodynamic diameter determined via DLS.

### 3.2. Characteristics of CO–HA Gels

**Figure 2:**
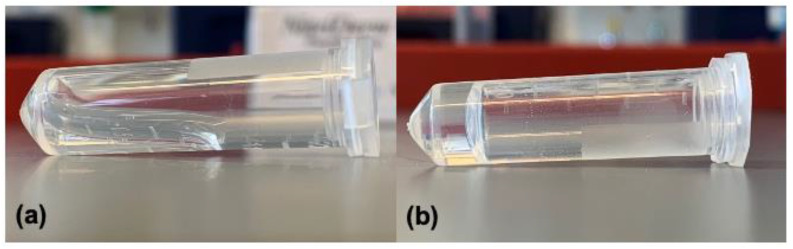
Representative photos of the blank hydrogel mixture at room temperature (**a**) and after incubation at 37 °C (**b**).

Two 8-arm PEG’s were used to prepare our hydrogel Gel 1 (MW: 20 kDa) and Gel 2 (MW: 40 kDa), respectively. It can be seen in Figure 2a that the CO–HA Gel mixture was in a liquid state at room temperature. Following incubation at 37 °C (body temperature), the mixture quickly crosslinked between succinimidyl groups of 8-arm PEG and primary amine groups of collagen to form a transparent semisolid gel (Figure 2b). The developed CO–HA Gel with good luminous transmittance (>98%) that was close to water (Figure S1a). To fabricate NP-loaded hydrogel composite, NP was mixed into the CO–HA Gel mixture prior to the gelation process. As shown in Figure 1b, NP with a spherical shape was homogeneously distributed in the CO–HA Gel composite matrix. The optical properties of NP incorporated CO–HA Gel composites was also studied (supplementary materials). The presence of a high dose NP in the NP/Gel composites resulted in a decrease in the luminous transmittance compared to the control (DI water) and CO–HA Gel group (Figure S1a). The luminous transmittance of the Cur NP/Gel composite between 380 and 480 nm was decreased, as curcumin has light absorbance at around 430 nm, but it was higher than that of the BLK NP/Gel composite when the wavelength was above 500 nm. Whether the presence of yellowish curcumin would potentially impair the visual process will be further investigated and discussed following an in vivo anti-UM dose optimization study in the future. Nevertheless, the Gel and NP/Gel composites studied can maintain good transparence as shown in Figure S1b.

#### 3.2.1. Rheological Properties

**Figure 3:**
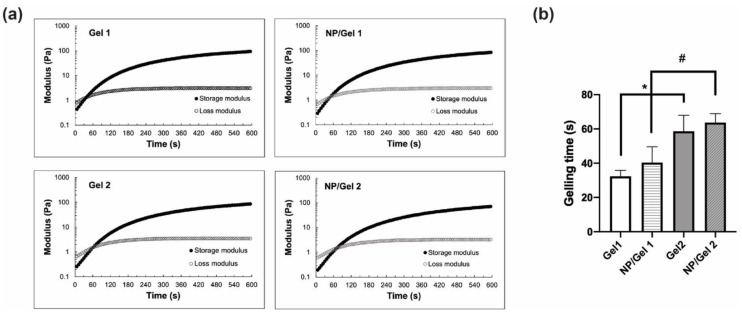
(**a**) Rheological properties of Gel 1, Gel 2 and nanoparticles (NP)/Gel composites obtained via time sweep with 1 Hz frequency and 5% strain at 37 °C (*n* = 3). (**b**) Gelling time of the different Gel and its NP/Gel composite (*n* = 3). Statistical analysis comparing between Gels or NP/Gel composites, *, # *p* < 0.05.

The gelling time of blank CO–HA Gels was determined using a time sweep at 37 °C. Both CO–HA Gel 1 and Gel 2 showed a short gelling time around or below 1 min at 37 °C. This confirmed that the developed CO–HA Gel can quickly transform from a liquid state to a semisolid gel state at body temperature. The CO–HA Gel 1 with a lower MW PEG had a shorter gelling time (32.37 ± 3.44 s) than that (58.66 ± 9.33 s) of the CO–HA Gel 2 with a higher MW PEG (Figure 3a,b). The same amount of collagen can crosslink with low MW PEG with a higher number of reactive groups in shorter duration [26]. The presence of Cur NP resulted in a slight increase in gelling time, but the impact was not significant. The NP/CO–HA Gel 1 and NP/CO–HA Gel 2 composites had a gelling time of 40.39 ± 9.21 and 63.64 ± 5.21 s, respectively (Figure 3b). Overall, Gel 1 and NP/CO–HA Gel 1 composite had a significant shorter gelling time compared with Gel 2 and NP/CO–HA Gel 2 composite (*p* < 0.05), respectively.

**Figure 4:**
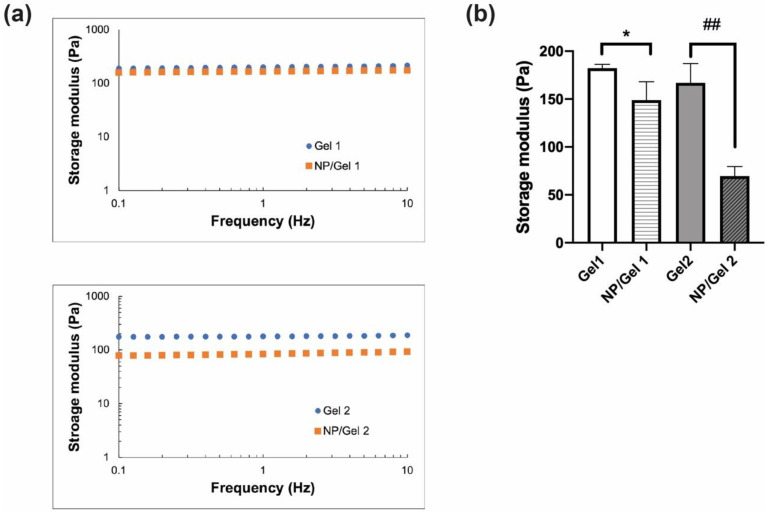
(**a**) Unconfined compression measurements of Gels and nanoparticle (NP)/Gel composites with oscillation frequency ranging from 0.1 to 10 Hz at 5% strain at 37 °C (*n* = 3). (**b**) The storage moduli of the Gels and their respective NP/Gel composites obtained at 5% strain and 1 Hz frequency (*n* = 3). Statistical analysis comparing between Gels and their respective NP/Gel composites, * *p* < 0.05, ## *p* < 0.01.

The mechanical properties of CO–HA Gels and NP/Gel composites were studied using unconfined compression testing. All the testing samples were evaluated in a LVR (0.5% to 20% strain). The average storage moduli of Gel 1 and Gel 2 determined based on the oscillation frequency method were 182.26 ± 3.93 and 166.84 ± 20.26 Pa (*p* > 0.05), respectively (Figure 4a,b). The presence of NP had a stronger effect on the storage modulus (69.54 ± 10.10 Pa) of the NP/Gel 2 composite, compared to that (148.96 ± 19.15 Pa) of the NP/Gel 1 composite. The crosslinking density of Gel or its NP/Gel composite was calculated based on their storage moduli. Gel 2 had a slightly lower crosslinking density (0.064 ± 0.008 mol/cm^3^) than Gel 1 (0.071 ± 0.002 mol/cm^3^), which can be explained by the less functional succinimidyl groups present in the higher MW PEG. Overall, the presence of NP resulted in a decrease in crosslinking density for both NP/Gel 1 and NP/Gel 2 composites (0.058 ± 0.007 mol/cm^3^ and 0.027 ± 0.004 mol/cm^3^, respectively). The presence of NP in the Gel mesh matrix may increase distance between polymer chains, thus decreasing the storage modulus [50]. The Gel 2 composite with a higher MW had higher collagen mobility and less reactive groups [26]. The presence of NP may further inhibit crosslinking, resulting in significant decrease in the storage modulus of the Gel 2 composite.

Overall, storage moduli of the CO–HA Gels and NP/CO–HA Gel composites were in a range of 55 to 200 Pa, which was comparable to many soft tissues including the human vitreous body (0.24–14.5 Pa) [51] and similar to the reported hydrogels studied as an artificial vitreous body [52]. In addition, our CO–HA Gel system with high viscosity (22.6 ± 2.73 Pa·s and 10.9 ± 1.59 Pa·s at 37 °C for NP/CO–HA Gel 1 and Gel 2, respectively) can increase the viscoelasticity of the vitreous body is unlikely cause turbulent flow in the human vitreous humor and [51,53], thus reducing the risk of retinal re-detachment. This indicated that the developed in situ gelling hydrogels and NP/hydrogel composites may be well tolerated in these soft tissues. In vivo compatibility will be studied using an animal model in the future.

#### 3.2.2. Internal Structure and Morphology of CO–HA Gels

**Figure 5:**
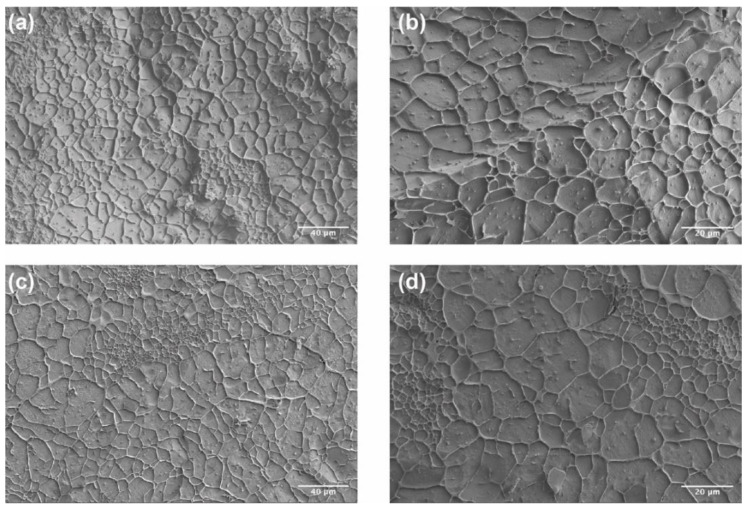
Cryo-SEM images of Gel 1 (20 kDa PEG) (**a**,**b**) and Gel 2 (40 kDa PEG) (**c**,**d**). Scale bar: (**a**,**c**) 40 μm; (**b**,**d**) 20 μm.

The internal structure of CO–HA Gel 1 and Gel 2 was characterized using cryo-SEM. As shown in Figure 5, Gel 2 showed a slightly smaller mesh area (70.66 ± 3.56%) and median mesh diameter (*D*_50_: 7.67 µm) than Gel 1 (mesh area: 73.70 ± 2.93% and *D*_50_: 9.89 µm, Table 2). In addition, Gel 2 had a higher mesh size span value (1.89) than Gel 1 (span value: 1.46), indicating that the meshes of Gel 2 had a wider mesh size distribution. It has been reported that the component and composition of a hydrogel and crosslinking density can influence hydrogel mesh size [28]. Considering that the difference in crosslinking density of Gel 1 and Gel 2 was not significant (*p* > 0.05), PEG MW appeared to play a significant role in the mesh size distribution. The high MW PEG with a longer polymer chain may lead to the formation of interpenetrating polymer networks and hence smaller meshes.

#### 3.2.3. Swelling Property and Equilibrium Water Content

**Figure 6:**
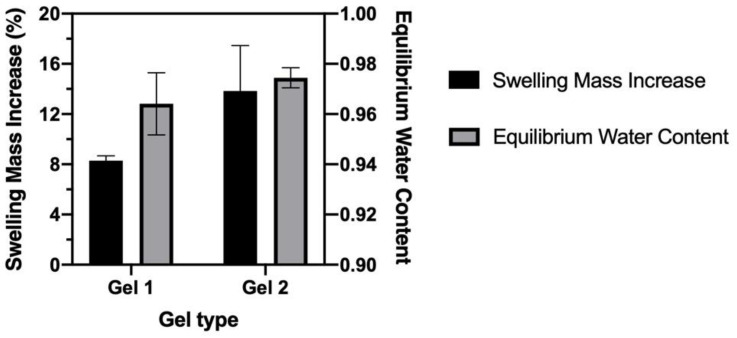
Swelling mass increase and equilibrium water content of CO–HA Gels (*n* = 3).

The swelling property and EWC of CO–HA Gels were also studied. Overall, our CO–HA Gels showed relatively low swelling (below 10%). Gel 1 with a higher crosslinking density exhibited less swelling (8.29 ± 0.37%) than Gel 2 (13.84 ± 3.61%) with a lower crosslinking density (Figure 6). The swelling of hydrogels inevitably causes decrease in mechanical strength, morphology changes, and bulk degradation [54,55]. In addition, swollen hydrogel may cause not only slippage from the implantation or injection site but also tissue compression [56]. Therefore, our CO–HA gel system with low swelling in the aqueous environment is considered desirable in achieving prolonged drug release while maintaining good compatibility with surrounding tissues. The calculated EWC of CO–HA Gel 1 and Gel 2 was 96 ± 0.12% and 97 ± 0.3%, respectively, which were very similar to that of HA hydrogel reported previously (EWC ranging from 60–99%) [57,58,59]. The water content of the human body is around 60% [60] and that of the eye vitreous body is around 98% [61]. Accordingly, our CO–HA Gels with similar fluid content to living tissues may have excellent tissue compatibility.

### 3.3. In Vitro Degradation of CO–HA Gels

**Figure 7:**
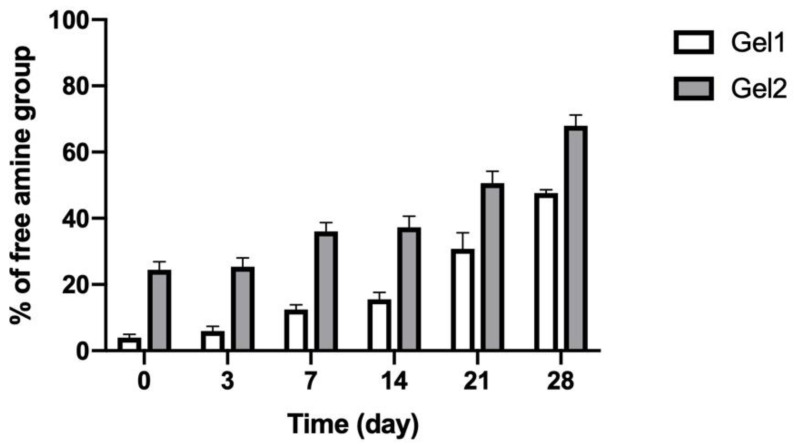
Percentage (%) of free amine groups in CO–HA Gels during in vitro degradation testing in saline at 37 °C over four weeks (*n* = 3).

In vitro degradation behavior of CO–HA Gel 1 and Gel 2 was studied in saline at 37 °C. CO–HA Gel was formed through ester linkers between PEG and collagen II and the ester linkers are susceptible to hydrolytic degradation. Therefore, the non-crosslinked free amine groups determined using the TNBS assay can indirectly reflect the crosslinking degree between PEG and collagen when compared with collagen alone [46,62]. The changes in free amine groups of Gel 1 and Gel 2 were monitored during a 4-week degradation study. The initial free amine group percentage of Gel 1 and Gel 2 without degradation was 3.9 ± 1.02% and 24.4 ± 2.45%, respectively. The lower amount of free amine groups in Gel 1 than Gel 2 confirmed the higher crosslinking degree of Gel 1 than Gel 2, which was consistent with the crosslinking density results described in Section 3.2.1. As shown in Figure 7, the percentage of free amine groups of both Gels gradually increased with the degradation of the hydrogels over 4 weeks. Gel 1 and Gel 2 had 47.6 ± 1.02% and 68.0 ± 3.21% free amine groups by day 28, respectively. There was no obvious gel structure change observed for both Gels. These results indicated that slow degradation behavior of the developed in situ CO–HA Gels, which can be used to achieve sustained payload release.

Collagenase and hyaluronidase are the main enzymes exist in the human body that can degrade collagen and hyaluronic acid, respectively. Therefore, in vitro degradation of CO–HA Gel was also studied in the presence of collagenase (75 U/mL) and hyaluronidase (16.7 U/mL). The high concentration collagenase can completely degrade Gel 1 and Gel 2 in 7 and 3 days, respectively. Under the neutral environments, the activity of hyaluronidase was largely decreased [63]. As a result, no obvious degradation was observed for Gel 1 and Gel 2 following a 7-day incubation of high concentration hyaluronidase (data now shown). It is worth mentioning that both collagenase and hyaluronidase are not abundant in the human vitreous humor [64,65]. Accordingly, the hydrolytic degradation may be the main degradation pathway of our CO–HA Gel. The degradation of CO–HA Gel in the vitreous humor will be studied using an animal model in the future.

### 3.4. In Vitro Release Characteristics of Cur NP/CO–HA Gel Composites

**Figure 8:**
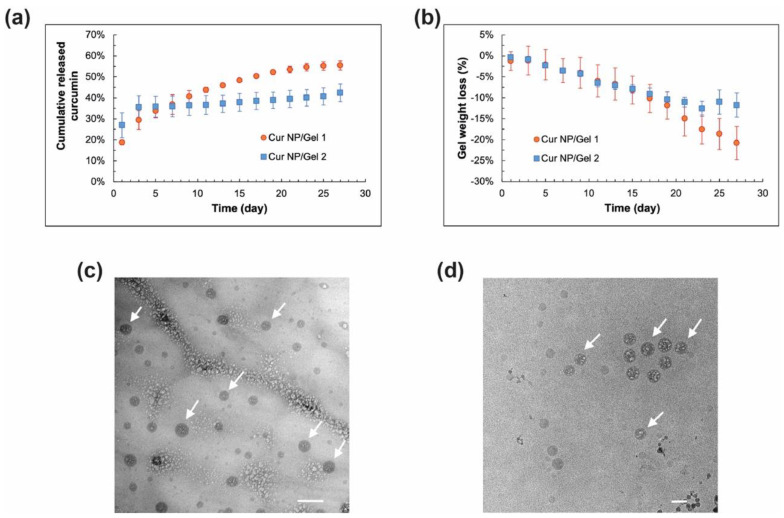
(**a**) In vitro curcumin release profiles and (**b**) weight loss profiles of curcumin nanoparticles (Cur NP)/Gel 1 and Cur NP/Gel 2 composites in saline containing 10% (*w*/*v*) Tween 80, 0.1% (*w*/*v*) N-acetylcysteine (NAC), and 0.01% (*w*/*v*) butylated hydroxytoluene (BHT) at 37 °C (*n* = 3). (**c**) Cryo-TEM image of the representative day-5 release sample of Cur NP/Gel 1 composite. (**d**) Cryo-TEM image of Cur NP dispersed in water as a control. Arrows indicate NP. Scale bar: 100 nm.

In vitro release characteristics of the Cur NP/Gel composites were studied in the presence of stabilizers including 10% (*w*/*v*) Tween 80, 0.1% (*w*/*v*) NAC, and 0.01% (*w*/*v*) BHT to minimize curcumin degradation during long-term release studies [66,67]. As shown in Figure 8a, curcumin slowly released from the Cur NP/Gel 1 composite and about 55% curcumin was released over four weeks, whereas the Cur NP/Gel 2 composite had a relatively higher curcumin release in the first two days, which may be due to higher swelling of Gel 2 and hence faster payload diffusion and higher initial curcumin release. With respect to the Cur NP/Gel 2 composite, curcumin release was slowed down after day 5 and reached a plateau in 10 days. The cumulative curcumin released was around 43% by the end of four weeks. Overall, the NP/Gel 1 composite showed better sustained curcumin release behavior. This may be attributed to its higher portion of large meshes through which payload can transport to the release medium (Table 2). In order to demonstrate whether intact NP can be released from the composite, release samples collected were imaged via Cryo-TEM. As shown in Figure 8c, spherical Cur NP was observed in the day-5 release samples, and the released Cur NP showed a similar particle size compared to that of the control group (NP suspended in water) (Figure 8d). These results demonstrated that intact Cur NP can be sustained released from our CO–HA Gels, which may lead to better transport of curcumin in the vitreous humor to reach its target posterior site (i.e., choroid).

The weight loss of the Cur NP/Gel composites was also monitored during the release study. It can be seen in Figure 8b that both NP/Gel composites had a similar weight loss over the first 20 days. The weight loss of the Cur NP/Gel 2 composite reached a plateau at around day 23, whereas a gradual weight loss was observed for the Cur NP/Gel 1 composite over four weeks. This was consistent with the sustained curcumin release from the Cur NP/Gel 1 composite. The Cur NP/Gel 1 composite with a better sustained curcumin release profile was selected for the anti-UM effect study.

### 3.5. In Vitro Anti-UM Effect of Cur CO–HA Gel Composites

**Figure 9:**
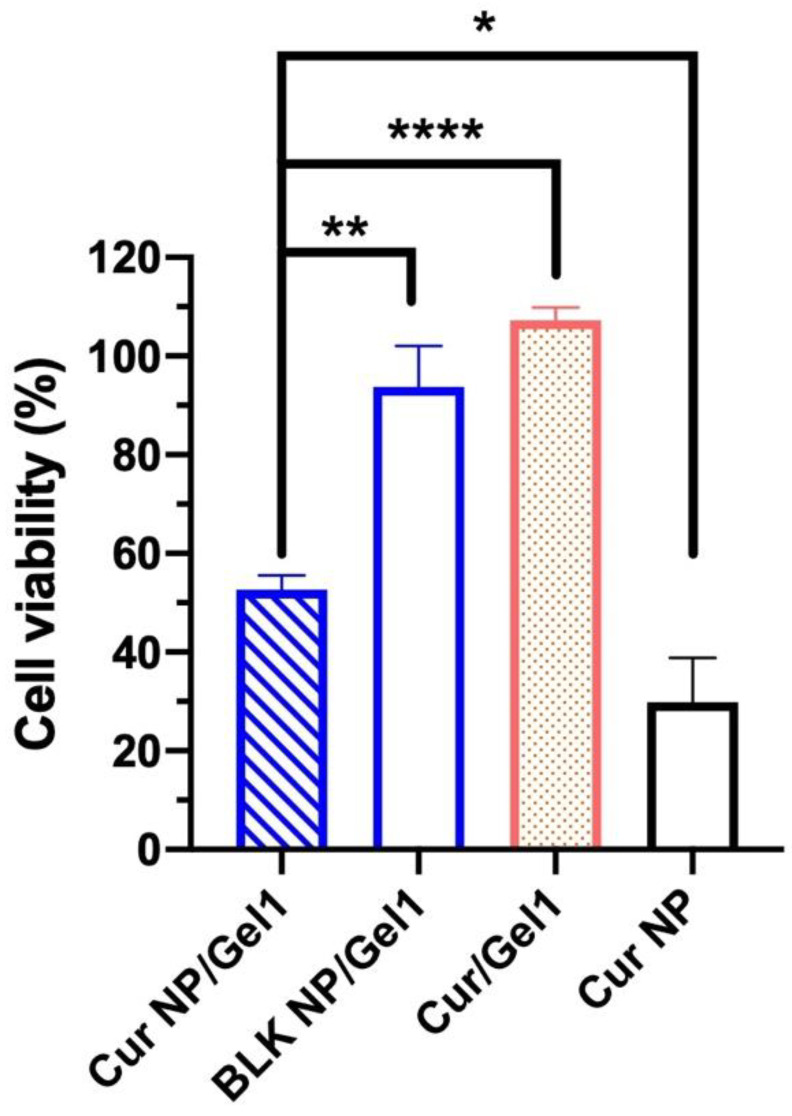
Cell viability of MP-38 cells following the treatment with curcumin nanoparticles (Cur NP)/Gel 1 composite, blank (BLK) NP/Gel 1 composite, Cur/Gel 1 composite and Cur NP for 3 days (*n* = 3). Statistical analysis comparing the Cur NP/Gel 1 composite with the control groups; * *p* < 0.05, ** *p* < 0.01, **** *p* < 0.0001.

A “proof-of-concept” short-term in vitro cell study for 3 days was conducted due to the challenges in culturing cells for prolonged duration (e.g., 30 days). Cur NP amount loaded into Gel 1 was determined based on curcumin IC_50_ and release behavior from the Cur NP/Gel composite (Figure 8a). Compared to the Cur/Gel 1 (cell viability: 107.3 ± 2.57%) and BLK NP/Gel 1 groups (cell viability: 93.8 ± 8.32%), the developed Cur NP/Gel 1 showed significantly decreased cell viability (52.7 ± 2.86%) (*p* < 0.01) (Figure 9). The result demonstrated that sustained curcumin release from the Cur NP/Gel 1 composite successfully inhibited UM cell growth. The poor anti-UM efficiency of the Cur/Gel 1 group may be resulted from the poorly water solubility and instability of curcumin in the aqueous environment (pH > 7) [68], thus the lack of sustained therapeutic effect [69]. The Cur NP group (NP alone) showed significantly lower cell viability (29.8 ± 9.04%) compared to the Cur NP/Gel 1 composite group, as the Cur NP amount was the same as that loaded in Cur NP/Gel 1 composite for a 30-day treatment instead of cur amount released in the first 3 days. It has been reported that curcumin can down regulate HIF-1α pathway [68]. A significant decrease in HIF-1α mRNA expression level of MP-38 cells following the treatment of Cur NP for 24 h under hypoxia conditions was observed via RT-qPCR (*p* < 0.05) (data not shown). This result suggested that downregulation of HIF-1α pathway could be one of the reasons for anti-UM effect of Cur NP.

**Figure 10:**
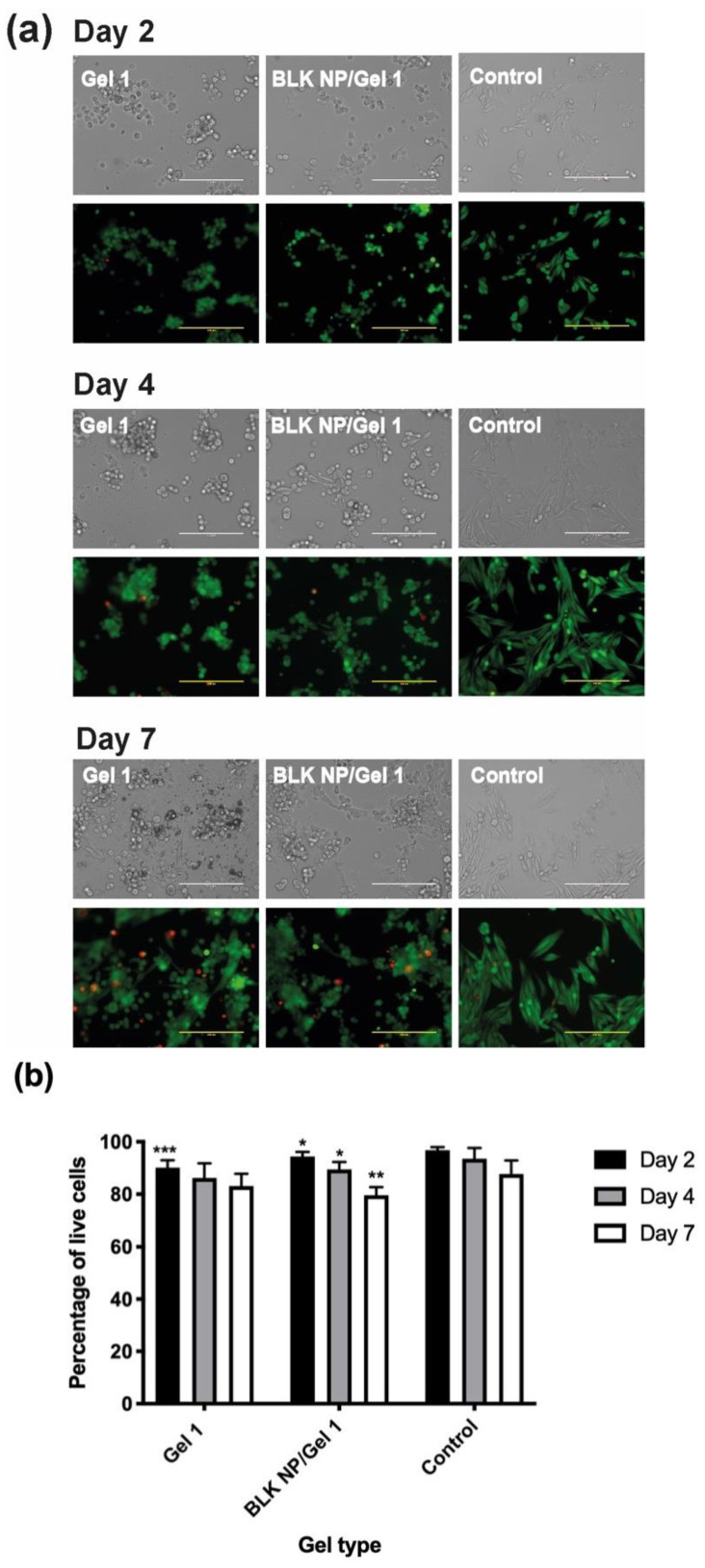
(**a**) Bright field and fluorescence images of MP-38 cells grown on CO–HA Gel 1 and blank NP (BLK NP)/Gel 1 composites at day 2, day 4, and day 7. MP-38 cells cultured in a 96-well culture plate were studied as a control. Scale bar: 200 µm. The MP-38 Cells were labeled with the LIVE/DEAD assay. Green and red fluorescence indicate live and dead cells, respectively. (**b**) Quantification results of live cell (%) of testing group: Gel 1, BLK NP/Gel 1 composites and the control (*n* = 3). Statistical analysis comparing experimental groups to the control group on the same day; * *p* < 0.05, ** *p* < 0.01, *** *p* < 0.001.

As shown in Figure 10, the MP-38 cells grown on the surface of the Gel 1 and BLK NP/Gel 1 composites had above 80% live cells during a period of 7 days, indicating low cytotoxicity. UM cells grown on culture plate was studied as a control and showed a significantly high live cell numbers compared to the Gel groups (*p* < 0.05) as culture plate has more even surface compared to hydrogel surfaces. It can be seen in Figure 10a that the cells grown on Gel 1 and BLK NP/Gel 1 composite had very similar morphology and live cell numbers (%), suggesting that the incorporated our PLGA NP did not decrease Gel’s cell compatibility. The low cytotoxicity of the CO–HA Gel 1 and its composite can be attributed to the excellent biocompatibility of the hydrogel components (i.e., collagen, HA). This result confirmed that the excellent anti-UM effect showed in Figure 9 was attributed to sustained Cur release from the Cur NP/Gel composite. Despite that the hydrogel components (i.e., collagen and hyaluronic acid) have excellent biocompatibility and good ocular safety of curcumin has been previously demonstrated using cell lines such as human retinal pigmented epithelial (D407) cells [70], 661W and ARPE-19 retinal cells [71], the ocular safety of the developed Cur NP/Gel composite needs to be investigated using an animal model in the future.

## 4. Conclusions

The present research developed a long-acting, injectable, in situ gelling hydrogel delivery platform inspired by the nature components of the vitreous body. The in situ gelling hydrogel composed of nature occurring polymers collagen and hyaluronic acid demonstrated excellent biocompatibility and can gel within minutes at the body temperature. In addition, the in situ gelling hydrogel with low swelling and soft storage modulus degraded slowly and was capable of sustained release payload over four weeks. More importantly, the curcumin nanoparticle/in situ hydrogel composite demonstrated excellent anti-UM effect in a short-term anti-UM effect study. These results demonstrated the great potential of the developed nanoparticle/in situ hydrogel composite for sustained treatment of uveal melanoma in the posterior chamber. The biodistribution in ocular tissues (e.g., choroid and retina) and anti-UM effect and safety (e.g., tissue compatibility and intraocular pressure) of the developed Cur NP/Gel composite need to be further studied using an animal model.

## Figures and Tables

**Table 1:** Particle size, size distribution and surface charge of polymeric nanoparticles (NP) (*n* = 3).

Sample	Size (nm)	PDI	Zeta Potential (mV)
Blank (BLK) NP	161.9 ± 5.1	0.074 ± 0.031	−26.1 ± 2.0
Lyophilized BLK NP	159.7 ± 2.2	0.069 ± 0.024	−27.5 ± 1.0
Curcumin (Cur) NP	156.6 ± 1.5	0.056 ± 0.027	−27.9 ± 1.2
Lyophilized Cur NP	163.4 ± 1.0	0.070 ± 0.021	−31.2 ± 0.2

**Table 2:** Mesh size of Gel 1 and Gel 2.

Gel	Mesh Diameter (µm)				Mesh Area (%)
	*D*_10_	*D*_50_	*D*_90_	Span	
Gel 1	5.70	9.89	20.13	1.46	73.70 ± 2.93
Gel 2	3.84	7.67	18.36	1.89	70.66 ± 3.56

## Data Availability

Data sharing not applicable. No new data were created or analyzed in this study. Data sharing is not applicable to this article.

## Supplementary Materials

The following are available online at https://www.mdpi.com/article/10.3390/pharmaceutics13091335/s1, Figure S1: (a) Optical transmittance spectra and (b) photo of CO–HA Gel 1 and NP/Gel 1 composites in a 96-well plate with a 125 µm sample thickness. Water was studied as a control. References [72,73].
