# Supplementary material for: A Long-Acting Curcumin Nanoparticle/In Situ Hydrogel Composite for the Treatment of Uveal Melanoma
Source: Pharmaceutics. 2021 Aug 25;13(9):1335. doi: 10.3390/pharmaceutics13091335 (PMC8467666; doi:10.3390/pharmaceutics13091335)
Supplement: Supplementary file 1 [file pharmaceutics-13-01335-s001.zip › pharmaceutics-1290894-supplementary.pdf]

pharmaceutics

Supplementary Materials: A Long-Acting Curcumin Nanoparticle/In Situ Hydrogel Composite for the Treatment of Uveal Melanoma

Lingxiao Xie, Weizhou Yue, Khaled Ibrahim and Jie Shen

Optical properties of CO-HA Gel and NP/CO-HA composites

The developed Gel 1 and NP/ Gel 1 composites) (40 µL) were placed in a 96-well plate and incubated at 37 °C to form hydrogels. The transmittance spectra of the samples were determined using SpectraMax iD3 (Molecular Devices, LLC., San Jose, CA, USA) in a wavelength range of 300–800 nm. Deionized water (40 µL) was studied as a control. The data was analyzed using SoftMax Pro 7.1 (Molecular Devices, LLC., San Jose, CA, USA).

The optical property (% light transmission) of CO-HA Gel 1, BLK NP or Cur NP/CO-HA composites (thickness: 125 µm) were compared with that of DI water [72]. Excellent luminous transmittance (above 98%) and transparence were observed for our CO-HA Gel 1, which was similar to that of water (Figure S1). The presence of a high dose NP in the NP/Gel composites resulted in decreased light transmittance. The lowest transmittance of BLK NP/Gel 1 was around 70% at 380 nm. Yellowish curcumin has light absorbance at around 430 nm, which was responsible for the decreased transmittance between 380 to 480 nm wavelength. The optical property between the 380-500 nm wavelength was compromised due to the presence of NP and curcumin. Nevertheless, the transparence of Cur NP/Gel 1 and BLK NP/Gel 1 was well maintained at a sample thickness of 125 µm as shown in Figure S1b. Overall, the developed NP/Gel composites had higher than 80% transmittance when wavelength was above 500 nm (the maximum transmittance through an entire eye is around 83.5% in this wavelength range [73]). Whether the presence of yellowish curcumin would potentially impair the visual process will be further investigated and discussed following an in vivo anti-UM dose optimization study in the future.

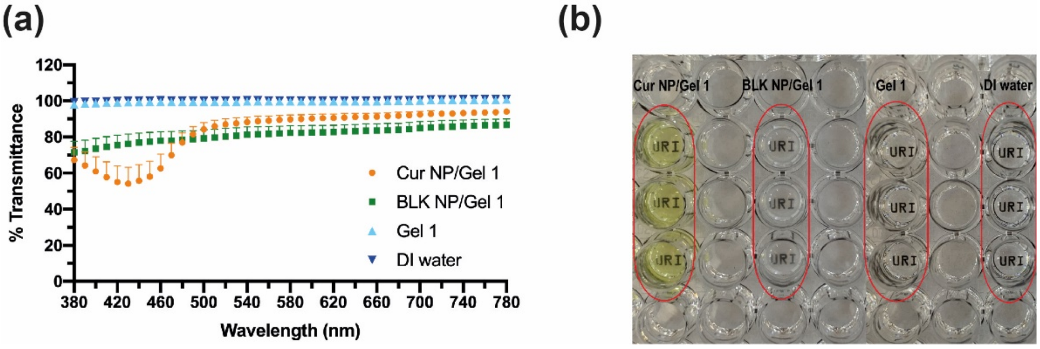

Figure S1. (a) Optical transmittance spectra and (b) photo of CO-HA Gel 1 and NP/Gel 1 composites in a 96-well plate with a 125 µm sample thickness. Water was studied as a control.

References

72. Zhou, Y.; Cai, Y.; Hu, X.; Long, Y. Temperature-responsive hydrogel with ultra-large solar modulation and high luminous transmission for “smart window” applications. *J. Mater. Chem. A* **2014**, *2*, 13550–13555.
73. Boettner, E.A.; Wolter, J.R. Transmission of the ocular media. *Investig. ophthalmol. & vis. sci.* **1962**, *1*, 776–783.
